# High-Precision Calibration of a Monocular-Vision-Guided Handheld Line-Structured-Light Measurement System

**DOI:** 10.3390/s23146469

**Published:** 2023-07-17

**Authors:** Jingbo Zhou, Zhaohui Ji, Yuehua Li, Xiaohong Liu, Wenhao Yao, Yafang Qin

**Affiliations:** 1 School of Mechanical Engineering, Hebei University of Science and Technology, Shijiazhuang 050018, China; zhoujingbo@hebust.edu.cn (J.Z.);; 2 Tangshan Yinglai Technology Co., Ltd., Tangshan 063000, China

**Keywords:** three-dimensional measurement, line structured light, handheld scanning, improved multi-view calibration

## Abstract

Due to the advantages of simple construction, easy application and good environmental suitability, handheld structured-light measurement systems have broad application prospects in 3D measurements. Here, a monocular-vision-guided line-structured-light measurement system is developed, and the posture of the handheld device can be obtained via a specifically designed target attached to it. No more marker points need to be adhered onto the object under inspection. The key for the system calibration is to obtain the coordinate transformation matrix from the sensor to the featured target coordinate system. The mathematical model of the system is first established. Then, an improved multi-view calibration method is proposed, where a selection process for the image pairs is conducted for accuracy improvement. With this method, the maximum relative error of the measured stair heights can be reduced from 0.48% to 0.16%. The measurement results for the specific parts further verified the effectiveness of the proposed system and the calibration method.

## 1. Introduction

Line-structured-light sensors (LSLSs) are based on the optical triangulation principle. They generally consist of a camera, a laser line projector and a frame that connects them together [1,2]. In the measurement process, a laser plane that emits from the laser line projector intersects the surface under inspection and a perturbed stripe that carries the profile information can be captured by the camera [3,4]. Since the relative position between the laser plane and the camera is fixed, the point coordinates on the intersection profile can be calculated through the pre-calibrated sensor parameters [5].

To achieve the measurement of three-dimensional (3D) surfaces, the LSLS needs to be integrated with other motion coordinates [6,7,8]. According to different measurement requirements, the type and number of motion coordinates are varied. The sensor can be integrated with a linear stage [9], a rotational table [10], a combination of linear and rotation motion stages [7], robotic arms [8,9] and an articulated arm measurement system for portable measurement applications [11,12,13]. However, the measurement range is limited by the motion range or the structural size of the mechanical systems. The integrity is affected by the degree of motional freedoms. Measurement accuracy also highly depends on the motion accuracy of the mechanical devices.

Compared with the motional-axes-based line-structured-light measurement systems (LSLMS), the handheld measuring devices are more flexible. The objects can be scanned from different viewpoints to ensure measurement integrity [14,15,16], although the accuracy of the result highly relies on the data fusion techniques and has the problem of unsmooth edges [17,18]. Data fusion via marker points can lead to a higher accuracy and has been widely adopted by the commercial handheld scanners [19]. Users need to adhere a sufficient number of highly reflective marker points onto the object; this is inconvenient. Another choice is to obtain the posture of the handheld device by use of an inertial measurement unit (IMU) [20,21]. The posture and the acceleration data from the IMU are greatly affected by external forces, vibrations and environments. It is difficult to achieve a high-precision measurement result with commercial IMU components.

Ayaz et al. [22] studied a 3D handheld scanning system based on visual–inertial navigation and structured light. This system had a limited scanning range and could only use block-matching algorithms for 3D reconstruction, resulting in low accuracy. Additionally, the introduction of inertial sensors reduced the scanning efficiency. Wang et al. [23] proposed a handheld laser scanning system for the on-site measurement of large objects. To collect the data points near the edges and highly curved areas of the object, it was required to paste density markers onto the object. Peng et al. [24] introduced a mechanical structure for an LSLS and a portable box volume measurement system based on deep learning. However, it needed strict calibration requirements and exhibited significant uncertainty when measuring the different surfaces of a box. These handheld devices require the attachment of markers onto objects for point cloud stitching. However, pasting marker points onto the object is inconvenient and sometimes not allowed. Here, a handheld LSLMS based on a non-planar feature target is presented to make the measurement task more convenient.

System calibration of the handheld measurement system can be summarized as a hand–eye problem. Currently, this problem can be solved based on the quaternion algebra [25], the Euclidean group [26], the dual quaternion [27], the Kroenke product [28,29], etc. Besides these, Cao et al. [30] combined neural networks with robot joint angles to compensate for non-geometric errors. Guo et al. [31] optimized the hand–eye relationship through the least squares method and the particle swarm optimization algorithm. Pachtrachai et al. [32] optimized the hand–eye matrix by the rotation translation matrix obtained by alternating iteration. These calibration methods are based on the fact that the initial position and pose have small deviations from the ideal value. Moreover, the original hand–eye data for the optimization process show a significant impact on the final calibration results. The method of how to reject unfavorite data still needs to be analyzed.

In this paper, a high-precision calibration method for handheld LSLMS is proposed by use of multi-view image pairs. Large noise images are eliminated through the data selection. The method improves the calibration accuracy of the coordinate transformation relationship between the sensor and the featured target coordinate system, leading to a better measurement result.

## 2. Mathematical Model

**Figure 1:**
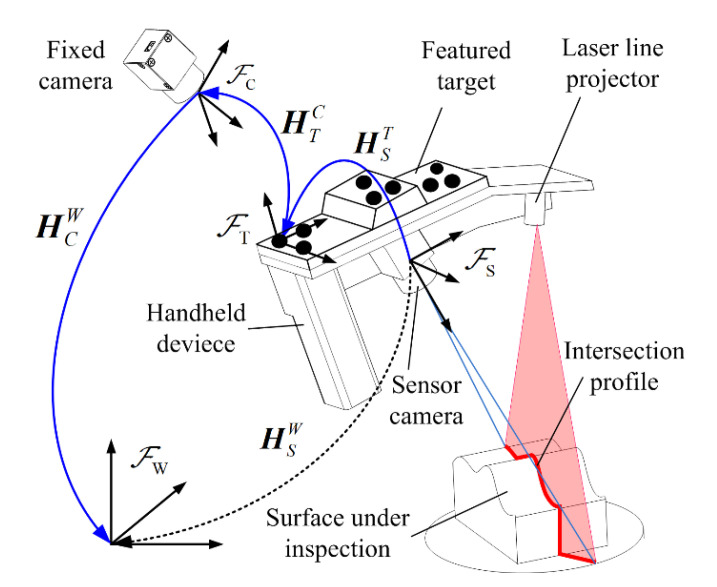
Illustration of the measurement system.

The proposed monocular-vision-guided LSLMS is shown in Figure 1. This system consists of a laser line projector, two cameras and a featured target. The laser line projector and one camera are fixed onto a handheld device, constituting an LSLS. With this sensor, an arbitrary intersection profile between the laser plane and the object under inspection can be obtained within the sensor coordinate frame ℱ_S_. The other camera is fixed in the world coordinate frame ℱ_W_ and captures the image of the featured target attached to the handheld device. ℱ_C_ and ℱ_T_ denote the fixed camera coordinate frame and the featured target coordinate frame, respectively. The posture of the handheld device can be computed in a real-time manner. By transforming the obtained profiles from ℱ_S_ to ℱ_T_ and then to ℱ_C_, we can obtain a series of intersection profiles on the object surface.

Assuming ***P****_s_* = (*X_s_*, *Y_s_*, *Z_s_*, 1)^T^ is a point on the intersection profile with the sensor coordinate values, ***P****_w_* = (*X_w_*, *Y_w_*, *Z_w_*, 1)^T^ is its corresponding coordinates within ℱ_W_, then

(1) $$P_{W}=H_{C}^{W}H_{T}^{C}H_{s}^{T}P_{s}$$

where $H_{S}^{T}$, $H_{T}^{C}$ and $H_{C}^{W}$ represent the coordinate transformation matrix and are denoted in Figure 1. $H_{T}^{C}$ can be computed in real time when the intrinsic parameters of the fixed camera and the dot interval distances on the feature target are known. $H_{C}^{W}$ only affects the relative position of the measurement results and has no influence on the geometrical accuracy. Thus, the key issue for system calibration is to obtain the value of $H_{S}^{T}$.

## 3. Calibration Method

### 3.1. Common Field-of-View Calibration Method

**Figure 2:**
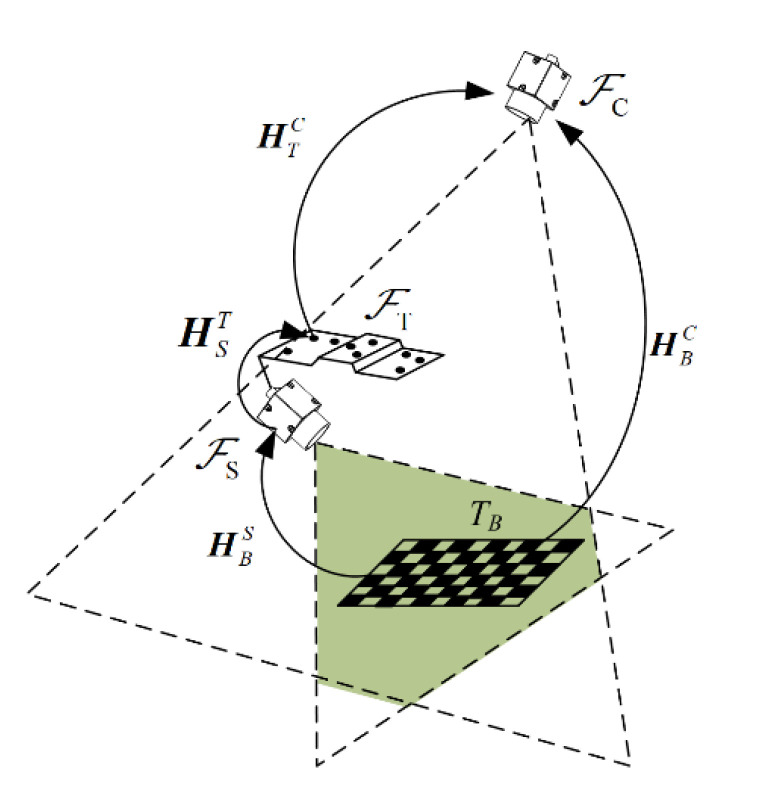
Common field-of-view calibration method.

The common field-of-view calibration method (CFVCM) is shown in Figure 2. A chessboard target *T_B_* is placed within the overlapped visual field of two cameras. The featured target on the handheld device is also located in the visual field of the fixed camera. Then, the transformation matrix from the sensor camera to the featured target can be expressed by

(2) $$H_{S}^{T}={\left(H_{T}^{C}\right)}^{-1}H_{B}^{C}{\left(H_{B}^{S}\right)}^{-1}$$

where $H_{B}^{C}$ and $H_{B}^{S}$ are the coordinate transformation matrices from the chessboard target to the sensor and the fixed camera, respectively. Both of them can be calculated via the intrinsic parameters and the known target parameters.

**Figure 3:**
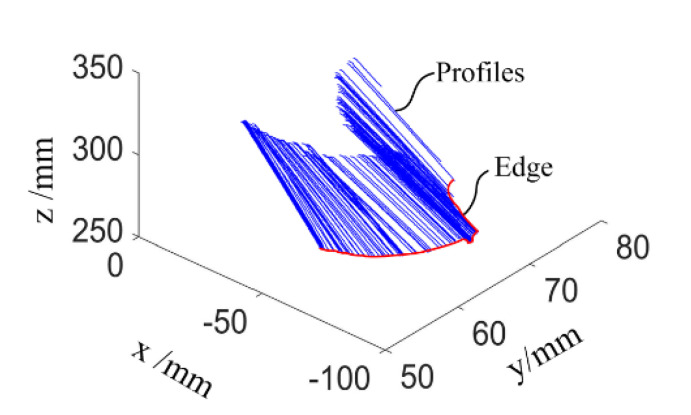
Measurement result for a plane with the CFVCM.

With this calibration method, a plane with a size of 60 × 60 mm is scanned, and the measurement point cloud is shown in Figure 3. The result is totally different from what we expected. Although all the intersection profiles are straight lines, they align in a disordered manner. The edge of the plane should also be a straight line. Now, it becomes an irregular space curve.

This failure result is most probably introduced by an inaccurate calibration result for $H_{S}^{T}$, which occurred for the following reasons. To achieve this calibration, both the planar target and the featured target should be located within the visual field of the fixed camera. The calibration target *T_B_* should also have a reasonable distance from the sensor camera to ensure image quality. Therefore, a significant difference exists between the planar and the featured target in the optical axis direction of the fixed camera. Additionally, this direction is also the error sensitive direction for the extrinsic parameter computation of $H_{B}^{C}$ and $H_{B}^{S}$. This is why the calibration results of $H_{S}^{T}$ cannot meet the accuracy requirements of the system.

### 3.2. Improved Multi-View Calibration Method

**Figure 4:**
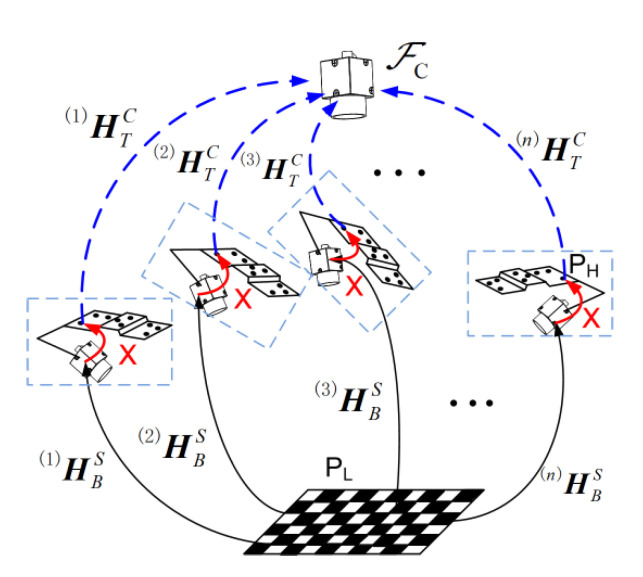
Multi-view calibration method.

To improve the calibration accuracy of $H_{S}^{T}$, an improved multi-view calibration method (MVCM) is proposed, as shown in Figure 4. For easy illustration, $H_{S}^{T}$ is represented by ***X***. ^(*n*)^$H_{T}^{C}$ stands for the posture of the featured target at the *n*th view of the handheld device; ^(*n*)^$H_{S}^{B}$ is the posture of the handheld camera that is computed via the chessboard underneath.

**Figure 5:**
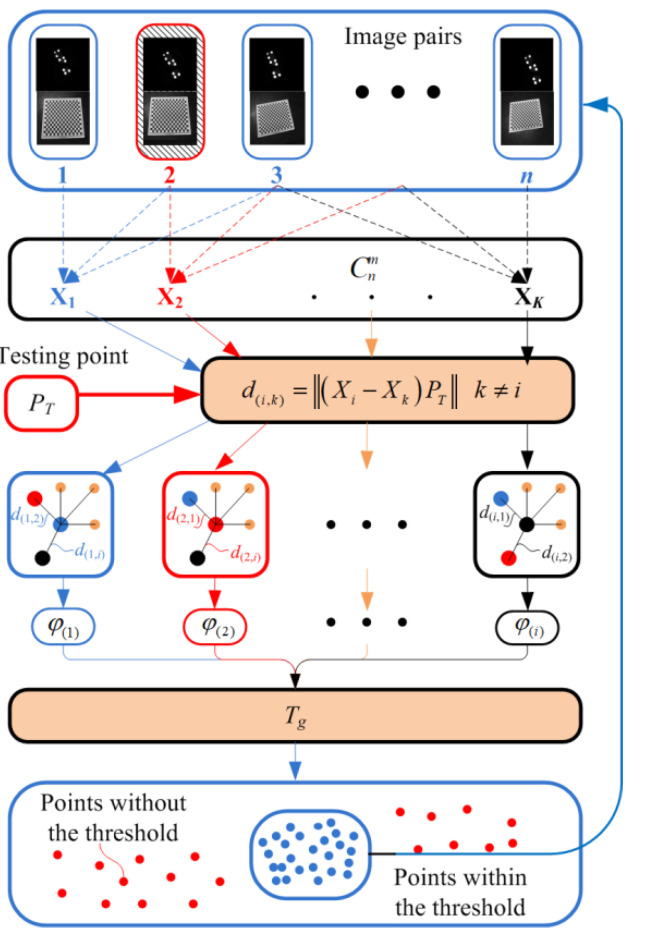
Procedures of image pair selection for calibration.

As the relative position between the chessboard and the fixed camera is unchanged, an arbitrary point on the chessboard has a constant coordinate value in the coordinate frame of ℱ_C_. Its coordinate value is irrelevant to the posture of the handheld device. The following equation can be achieved for two arbitrarily chosen positions of the handheld device:

(3) HTC(i)XHBS(i)=HTC(j)XHBS(j)

where ***X*** is the coordinate transformation matrix to be solved. From Equation (3), it can be seen that the essence of this problem is a hand–eye calibration problem in the robotic research area. The difference is the posture of the handheld device is computed by using the featured target attached to it, not the coordinate values of the robotic joints. Here, the coordinate transformation matrix ***X*** is calculated by the classical method brought by Park and Martin [26]. Then, a calibration strategy is brought out to identify and reject some image pairs that may deteriorate the accuracy of the calibration results. The procedures are illustrated in Figure 5.

For each position of the handheld device, two images need to be captured. One is the featured target image for posture computation of the handheld device. The other is the chessboard image that can be used to obtain $H_{S}^{B}$. These two images can be called an image pair. Assuming that the number of image pairs is *n*, *m* is the number that is chosen for the calibration with the least squares criterion [31]. $C_{n}^{m}$ combinations can be found. Each combination corresponds to one calibrated result denoted by ***X****_k_*. A virtual testing point method is brought out to detect the unfavorite image pairs. The measurement process is to obtain the measurement value in the world coordinate system with the transformation relationship shown by Equation (1). The calibration error of the matrix ***X****_k_* would make the translated point in the world coordinate system not be overlapped. The distances between the translated points are used to detect the unfavorite image pairs.

The selected image pairs are obtained by counting the number of the occurrence results that are within the threshold value. A more accurate calibration result of ***X*** can be achieved by a recalculation process using the selected image pairs. Assuming $P_{F}^{i}$ is the *i*th point in ℱ_C_ that is calculated by use of ***X****_i_*, the relative distances between this point and the rest of the points are denoted by ***D****_i_* = {*d*_(*i*,1)_, *d*_(*i*,2)_, …, *d*_(*i,k*)_,…, *d*_(*i,b*)_}, *b* = *C*_n_^m^− 1. The interclass variance of *d*_(*i*,*k*)_ within the data set of ***D****_i_* can be calculated by

(4) $$\sigma_{i}^{2}(d_{\left(i,k\right)})=\omega_{0}^{i}{\left(\mu_{0}^{i}-\mu_{T}^{i}\right)}^{2}+\omega_{1}^{i}{\left(\mu_{1}^{i}-\mu_{T}^{i}\right)}^{2}$$

where $w_{0}^{i}$ *= l*/(*b* − 1), *l* is the number of the elements that are smaller than *d*_(*i,k*)_ in the data set of ***D****_i_*, $w_{1}^{i}$ = 1 − $w_{0}^{i}$ denotes the proportion of other distance values, μ_0_ is the average value that is smaller than *d*_(*i,k*)_, μ_1_ is the average value of the elements that are larger or equal to *d*_(*i,k*)_ and *μ*_T_ is the average value of all the elements of ***D****_i_*. When $\sigma_{i}^{2}$(*d*_(*i,k*)_) achieves its maximum value, its corresponding value of *φ*_(*i*)_ is the optimal threshold value for the data set ***D****_i_*.

(5) $$f\left(\varphi_{\left(i\right)}\right)=f\left(\underset{1\leq k\leq b}{max}\left\{\sigma_{i}^{2}\left(d_{\left(i,k\right)}\right)\right\}\right)$$

Similarly, we can obtain a distance data set for an arbitrary point within the fixed camera coordinate system. Then, its corresponding optimal threshold value can be achieved using Equations (4) and (5). The optimal threshold values for different points can generate a new data set and are denoted by ***Ψ****_i_* = {*φ*_(1)_, *φ*_(2)_, …, *φ*_(*i*)_}, *i =* 1,2, …, $C_{n}^{m}$. By computing the interclass variance of each value within the data set ***Ψ****_i_*, we can also find a maximum value. Its corresponding value of *φ*_(*i*)_ is the final threshold value for the distance segmentation.

(6) $$f\left(T_{g}\right)=f\left(\underset{1\leq i\leq C_{n}^{m}}{max}\left\{\sigma_{i}^{2}\left(\varphi_{\left(i\right)}\right)\right\}\right)$$

After that, each value of *d*_(*i,k*)_ can be compared with *T_g_,* and the ones that are larger than *T_g_* are deleted. We can count the image sequences corresponding to the selected distance values. The image pairs that have the lowest frequencies would be deleted. This process would be repeated until the optimal image sequences have been found. The rest image pairs are used to recalculated ***X*** using the hand–eye calibration method. The detailed system description and calibration procedures are as follows.

## 4. Calibration Procedures

### 4.1. Setup of the Measurement System

**Figure 6:**
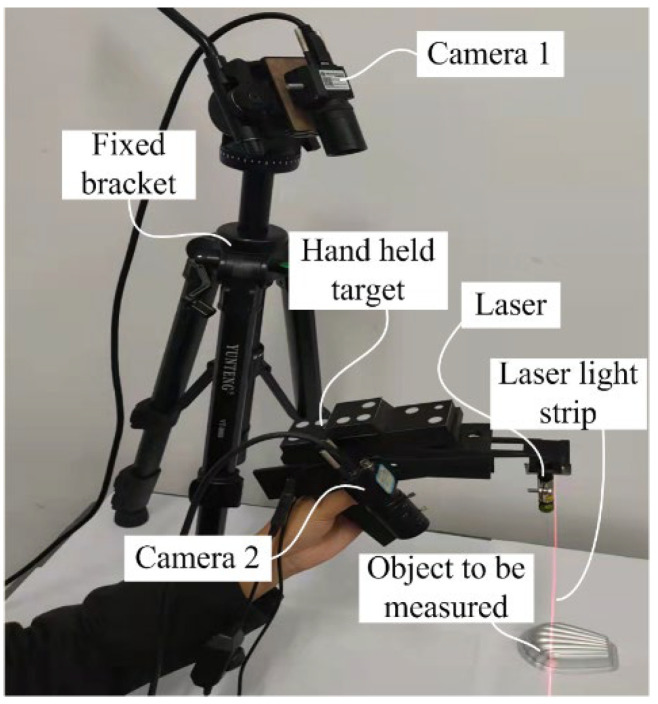
Prototype of handheld LSLMS.

The hardware of the handheld LSLMS is illustrated in Figure 6. In order to ensure the synchronization of the images taken by the handheld structured-light sensor camera and the vision guiding camera, global exposure cameras (Shenzhen Mindvision, MV-SUA134GM-T, Shenzhen, China) with an external trigger function were selected. The wavelength range of the sensitive spectrum of the camera’s CMOS detector was 400 nm to 950 nm, covering the spectral content of the feature target and laser. The cameras had 1280 × 1024 pixels with an image acquisition frequency of 211 fps, which could meet the requirements of the handheld scanning situations. The wavelength of the laser was 632.8 nm (Guangdong Shengzuan Lasers Co., Ltd., Shenzhen, China), and the minimum stripe width could reach 0.3 mm at a projection distance of 300 mm.

**Figure 7:**
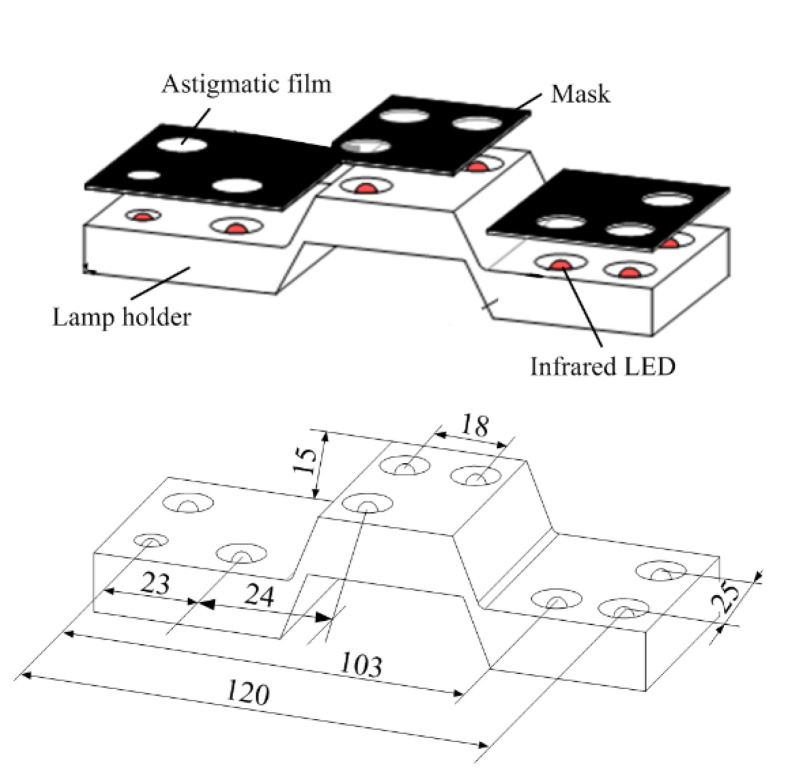
Composition and key parameters of featured target.

The featured target on the handheld device is also important to ensure measurement accuracy. The composition and key parameters of the target are shown in Figure 7. The light spot of the target was asymmetrically arranged to facilitate the sorting of spots. Independent infrared LEDs illuminated each spot with a wavelength of 850 nm. One side of the mask was coated with a light scattering layer, and the other side was processed with a circular hole corresponding to the lamp position to ensure the clarity of the feature points. The lamp holder was fabricated using a 3D printing technique. The position accuracy of the dots could not fulfill the measurement requirements. Therefore, a binocular method was used to recalibrate the relative distances between the feature points. The calibrated results were used for the computation of the extrinsic parameters.

### 4.2. Calibration Procedures

Calibration of the measurement system mainly included three steps. The first step was to calibrate the internal parameters of two cameras using Zhang’s method [33]. The next step was the sensor calibration where the equation of the laser plane needs to be computed in the handheld camera coordinate system [2]. The final step was to calibrate the transformation matrix between the featured target coordinate system and the handheld camera coordinate system. The camera and the sensor calibration methods are relatively mature. Here, we focus on the final step. To achieve the calibration, 15 chessboard and corresponding feature target images were taken at different postures of the handheld device. During this process, the laser line projector was turned off to ensure the image quality of the chessboard. Then, the calibration was completed using the method in Section 3.2.

**Figure 8:**
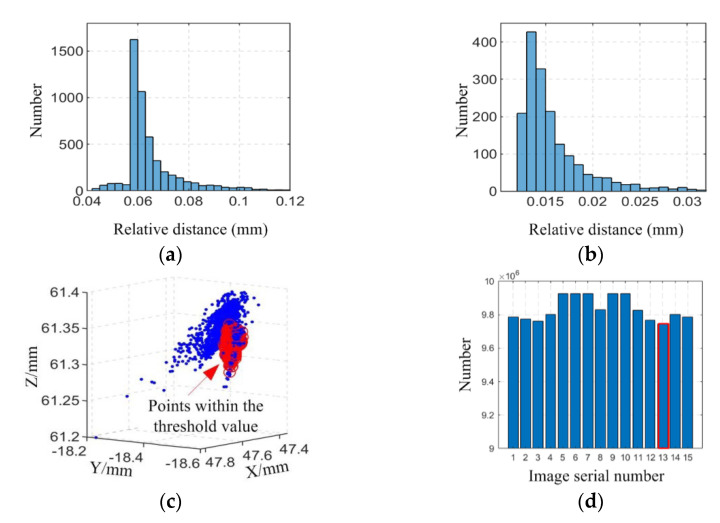
Selection of image pairs. (**a**,**b**) Relative distance of the distribution point cloud after transformation. (**c**) Point clouds before and after selection. (**d**) Image sequence.

Figure 8a,b represent the relative distances of the distribution point cloud after transformation. Figure 8c represents the distribution of point clouds before and after selection. Figure 8d represents the frequency of specific image pairs that are used for the computation of selected points. These points are within the threshold value of *T_g_*. The image pair that has the least frequency is rejected for this iteration.

Table 1 shows the change in the proportion of large noise points (***P****_n_*) within the data set of ***D****_i_* in the selection process of image pairs. For each iteration, one image pair would be deleted. Meanwhile, ***P****_n_* is also computed by comparing the number of points from *T_g_* and the total number. It can be seen from this table that ***P****_n_* first decreases and then increases. The reason is that as the images with large errors are eliminated, the amount of data deviating from the threshold in the current data set becomes smaller and smaller. When the image pairs that may introduce significant calibration errors are eliminated, the rest of the image pairs meet the pre-set threshold and are at the same numerical level. When the new threshold is computed, the proportion of large noise points from the new threshold in the remaining data will gradually increase. Therefore, when the iteration number is three, the calibration can reach the optimal result.

The value of $H_{S}^{T}$ can be obtained before and after the image selection process. For easy comparison, the rotation angles and the translation vectors are computed and are shown in Table 2. It can be seen that the image selection process has a significant impact on the calibration results. The value of *t_z_* has a variation of even more than 1 mm. The effect of the calibration matrix on the measurement accuracy can be found in Section 5.2.

## 5. Measurement Results and Discussions

### 5.1. Measurement of Typical Parts and Accuracy Evaluation

**Figure 9:**
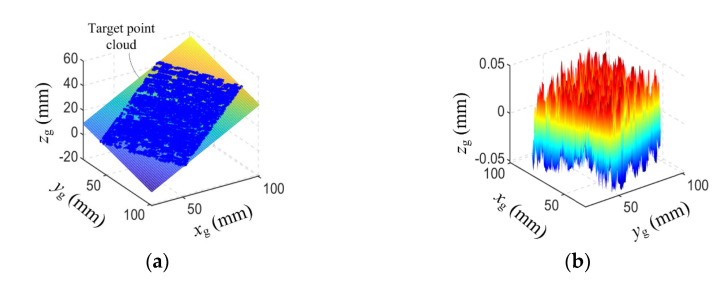
Measurement results of a plane. (**a**) Point cloud. (**b**) Residue error.

The plane has already been measured using the system that is calibrated by use of the CFVCM. It is a failure result, as shown in Figure 3. After calibration with the improved MVCM, the same plane is rescanned using the handheld LSLMS. The point cloud and the fitted plane are shown in Figure 9a. Nearly all of the points are located on the fitted plane and the edge of the point cloud now becomes straight. Some points are located under the fitted plane due to the least squares fitting criterion and cannot be seen. This is why some holes existed in the measurement result. When the tilt is removed, the residual surface can be seen, as shown in Figure 9b. The evaluation parameters of the measurement results can be obtained with a maximum deviation of 0.0523 mm. The absolute mean value is just 0.0285 mm, denoting a high measurement accuracy for the calibration method.

**Figure 10:**
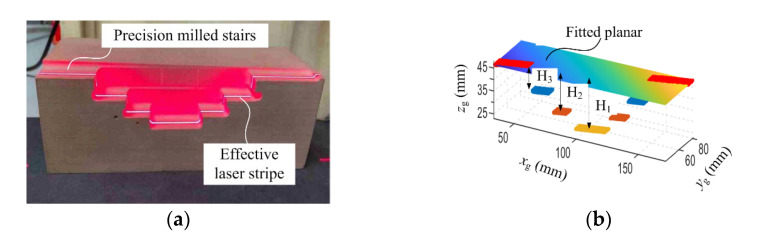
Measurement of the stairs. (**a**) Stairs. (**b**) Measured point cloud.

To further validate the effectiveness of the measurement system, a part with precisely milled stairs was measured, as shown in Figure 10a. The diffused laser stripe from the stairs can be seen clearly. This stripe is thin and bright, which ensures the accuracy of the results. Figure 10b is the measured point cloud and the height of each stair has been denoted by H_1_, H_2_ and H_3_. The fitted plane of the top stair is the reference for height computation.

To make the result more reasonable, the edge points of each stair have been removed. The height values are the mean distance between the points on the stair and the planar fitting. The height of the stairs obtained by the coordinate measuring machine (Hexagon Golbal 7107, Qingdao, China) is considered as the standard value, and they are H_1_ = 24.0039 mm, H_2_ = 18.0027 mm and H_3_ = 10.0021 mm. The measurement results from the use of the MVCM and the improved MVCM were compared. With each method, the stairs are scanned five times. The measurement errors, the absolute mean error (AME) and the relative error (RE) are shown in Table 3 and Table 4.

The maximum relative error of the measured stairs with the handheld structured-light equipment calibrated using MVCM is 0.48%. With the improved method, the maximum relative error is reduced to 0.16%.

**Figure 11:**
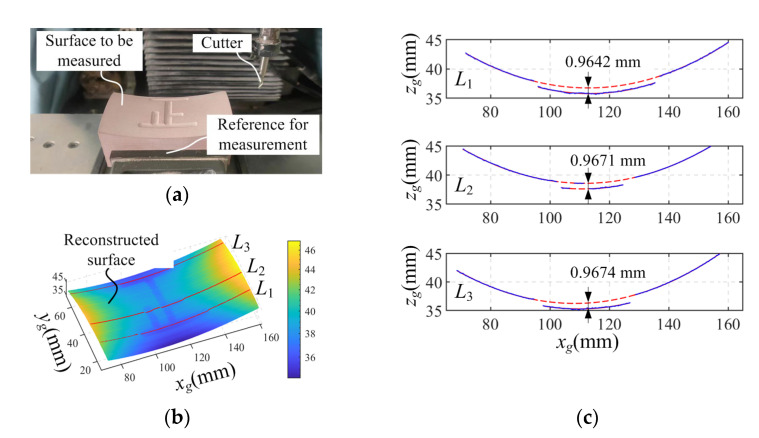
Measurement and accuracy verification of a complex surface. (**a**) Surface to be measured, (**b**) reconstructed surface, (**c**) the intersection curves measured by the handheld device.

Besides the plane and the stairs, we also milled a more complex surface on a precision machine, as shown in Figure 11a. It is a saddle surface with an equation in the workpiece coordinate system of:

(7) $$z_{w}=\frac{{\left(x_{w}-42.5\right)}^{2}}{200}-\frac{{\left(y_{w}-27.5\right)}^{2}}{200}-8$$

where *x_w_*, *y_w_* and *z_w_* are the 3D coordinate values of the surface points. The range of values for *x_w_* and *y_w_* are 0–85 mm and 0–55 mm, respectively. A character is engraved in the surface with a programming depth of 1 mm. After machining, the handheld scanning device was used to obtain the point cloud data of the surface, and the reconstructed result is shown in Figure 11b. The reference surface is adopted for the coordinate alignment between the measurement system and the workpiece system.

Three cross-sectional profiles, *L*_1_, *L*_2_ and *L*_3_, are selected along the *x_g_* direction on the measured surface for analysis. According to Equation (7), the profiles of the saddle surface in these three sections are all quadratic curves. Therefore, the least squares method was employed to fit the data of these profile sections. The average distance between the data points on the milled character profile and the corresponding fitted curve was calculated as the measurement value of the milling depth, as shown in Figure 11c.

The depth of the character was also measured using the coordinate measuring machine, with the measurement points distributed on the surface and the bottom of the character. Equation (7) was used to fit the measurement data of the saddle surface, and the average distance between the detection points of the character section and the fitted surface was calculated as 1.0044 mm. This deviation from the programmed milling depth is 4.4 μm, which can be used as a reference value for the measurement results. By comparison, the deviations of the character depth from the reference values in the three cross-sections were 0.0402 mm, 0.0373 mm and 0.0370 mm, respectively.

From the experiments, it can be seen that the handheld scanning device has maximum errors of 0.0285 mm, 0.0243 mm and 0.0402 mm when measuring planes, stepped surfaces and complex curved surfaces, respectively. The measurement error is within 0.0500 mm. In comparison, Creaform’s handheld scanning system demonstrates a measurement accuracy ranging from 0.0250 mm to 0.0500 mm [34]. It should be noted that their measurement systems require binocular guidance (MetraSCAN 3D) or the use of marker points (the series of HandySCAN 3D). Our measurement system, on the other hand, achieves comparable accuracy through only monocular guidance and can be easily used without marker points. This indicates that the proposed calibration method enhances the accuracy significantly for monocular measurement systems.

### 5.2. Measurement of Parts with Complex Surfaces

**Figure 12:**
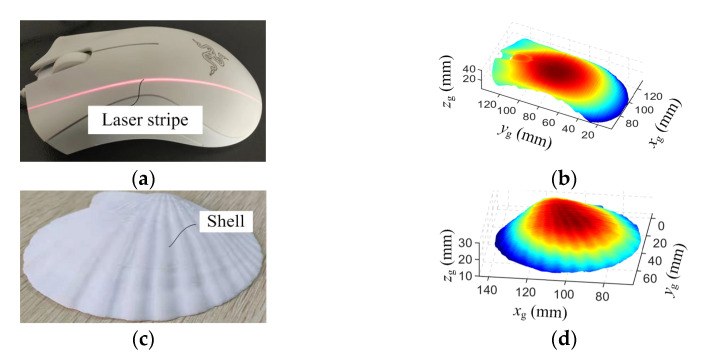

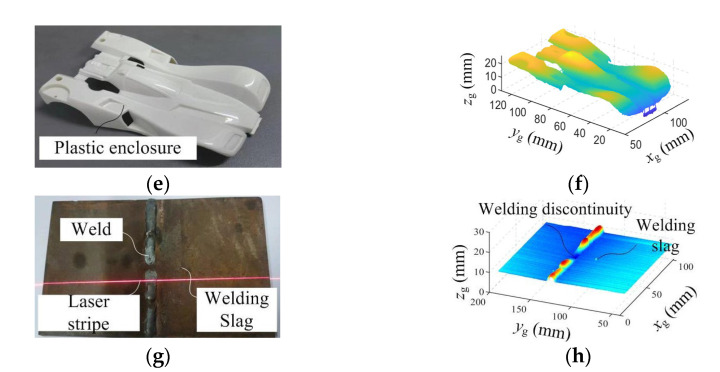
Measurement of typical parts. (**a**) The mouse, (**c**) the natural shell, (**e**) plastic enclosure and (**g**) the welded part; (**b**,**d**,**f**,**h**) are the reconstructed surfaces.

The aim of the measurement system is to obtain 3D geometrical information of the parts for quality inspection and reverse engineering. Therefore, a mouse, a natural shell, a plastic enclosure and a welded part were scanned, as shown in Figure 12a,c,e,g. With the proposed scanning system, these parts can be scanned from different angles. This makes the system more flexible and leads to a more integrated result. The measured point cloud is processed with the Delaunay triangulation method. Figure 12b,d,f,h show the triangulated surfaces.

Figure 12b presents the contour of the mouse and accurately depicts the details of the scroll wheel; Figure 12d clearly displays the texture of the seashell surface; Figure 12f demonstrates the measurement results of the toy car’s enclosure and accurately represents the concave and convex features; Figure 12h shows the reconstructed surface of the welded part, revealing welding discontinuity and providing the three-dimensional point cloud information of the weld slag. Therefore, this handheld scanning system shows potential for defect detection in mechanical manufacturing. The aforementioned scanning reconstruction results further validate the effectiveness of the handheld scanning device in capturing the 3D geometric information of complex surfaces.

## 6. Conclusions

A handheld LSLMS was developed for the 3D geometrical measurement of complex parts. This system is guided by monocular vision and can be applied without linear slides or rotation tables. It consists of a movable structured-light sensor and a pose measurement camera. High-precision calibration of the coordinate transformation matrix from the movable line-structured-light sensor to the handheld target coordinate system is the prerequisite for measurement. To achieve this goal, an improved MVCM is proposed together with a selection procedure for image pairs. The accuracy of the transformation matrix from the sensor to the feature target on the handheld device is improved. With this method, the maximum relative error of the measured stair heights can be reduced from 0.48% to 0.16%. The maximum measurement error for the saddle surface is 0.0402 mm. The proposed measurement system does not need to have pasted markers on the objects during scanning, thereby avoiding the errors and time consumption associated with point cloud stitching. Additionally, monocular guidance has lower usage requirements and cost, making it applicable for quality inspection and reverse engineering. The next step is to develop a real-time display and result evaluation module for in situ measurement.

## Figures and Tables

**Table 1:** Proportion of large noise points in the selection.

Iteration Number	1	2	3	4	5	6
***P* *_n_***	0.0905	0.0745	0.0633	0.1109	0.1227	0.1850

**Table 2:** Parameters of the calibrated matrix before and after image selection.

	*α* (°)	*β* (°)	*γ* (°)	*t_x_* (mm)	*t_y_* (mm)	*t_z_* (mm)
Before	0.3271	−0.0284	0.0149	68.1483	74.3070	−115.7414
After	0.3266	−0.0288	0.0131	68.8641	73.4806	−117.1400

**Table 3:** Measurement results using MVCM (mm).

No.	1	2	3	4	5	AME	RE
H_1_	−0.0564	0.0330	−0.0921	0.1244	0.1407	0.0893	0.37%
H_2_	−0.0418	0.0684	−0.0645	0.0554	0.0868	0.0634	0.35%
H_3_	−0.0656	0.0734	−0.0354	0.0289	0.0372	0.0479	**0.48%**

**Table 4:** Measurement results using improved MVCM (mm).

No.	1	2	3	4	5	AME	RE
H_1_	0.0030	−0.0139	−0.0339	−0.0196	−0.0223	0.0185	0.08%
H_2_	−0.0188	−0.0353	−0.0305	−0.0069	−0.0302	0.0243	0.13%
H_3_	−0.0080	−0.0034	−0.0320	−0.0039	−0.0319	0.0158	**0.16%**

## Data Availability

Data sharing not applicable.

## References

[1] Wei Z., Shao M., Wang Y., Hu M. (2013). A Sphere-Based Calibration Method for Line Structured Light Vision Sensor. Adv. Mech. Eng..

[2] Li Y., Zhou J., Mao Q., Jin J., Huang F. (2020). Line Structured Light 3D Sensing With Synchronous Color Mapping. IEEE Sens. J..

[3] Usamentiaga R., Garcia D. (2019). Multi-camera calibration for accurate geometric measurements in industrial environments. Measurement.

[4] Li Y., Zhao B., Zhou J., Ren Y. (2021). A universal method for the calibration of swing-scanning line structured light measurement system. Optik.

[5] Liang J., Gu X. (2020). Development and application of a non-destructive pavement testing system based on linear structured light three-dimensional measurement. Constr. Build. Mater..

[6] Karaszewski M., Sitnik R., Bunsch E. (2012). On-line, collision-free positioning of a scanner during fully automated three-dimensional measurement of cultural heritage objects. Robot. Auton. Syst..

[7] Logozzo S., Valigi M.C., Canella G. (2018). Advances in Optomechatronics: An Automated Tilt-Rotational 3D Scanner for High-Quality Reconstructions. Photonics.

[8] Babu M., Franciosa P., Ceglarek D. (2019). Spatio-Temporal Adaptive Sampling for effective coverage measurement planning during quality inspection of free form surfaces using robotic 3D optical scanner. J. Manuf. Syst..

[9] Li Y., Zhou J., Huang F., Liu L. (2017). Sub-Pixel Extraction of Laser Stripe Center Using an Improved Gray-Gravity Method. Sensors.

[10] Zhou J., Wang K., Yang G., Liu X., Du R., Li Y. (2022). Real-time uncertainty estimation of stripe center extraction results using adaptive BP neural network. Measurement.

[11] Santolaria J., Aguilar J.-J., Yagüe J.-A., Pastor J. (2008). Kinematic parameter estimation technique for calibration and repeatability improvement of articulated arm coordinate measuring machines. Precis. Eng..

[12] Santolaria J., Guillomía D., Cajal C., Albajez J.A., Aguilar J.J. (2009). Modelling and Calibration Technique of Laser Triangulation Sensors for Integration in Robot Arms and Articulated Arm Coordinate Measuring Machines. Sensors.

[13] Tai D., Wu Z., Yang Y., Lu C. (2023). A Cross-Line Structured Light Scanning System Based on a Measuring Arm. Instruments.

[14] Park S.-Y., Baek J., Moon J. (2011). Hand-held 3D scanning based on coarse and fine registration of multiple range images. Mach. Vis. Appl..

[15] Ayaz S.M., Kim M.Y. (2017). Multiview registration-based handheld 3D profiling system using visual navigation and structured light. Int. J. Optomechatron..

[16] Newcombe R.A., Izadi S., Hilliges O., Molyneaux D., Kim D., Davison A.J., Kohi P., Shotton J., Hodges S., Fitzgibbon A. KinectFusion: Real-time dense surface mapping and tracking. Proceedings of the 2011 10th IEEE International Symposium on Mixed and Augmented Reality.

[17] Han J., Shao L., Xu D., Shotton J. (2013). Enhanced Computer Vision with Microsoft Kinect Sensor: A Review. IEEE Trans. Cybern..

[18] Sarbolandi H., Lefloch D., Kolb A. (2015). Kinect range sensing: Structured-light versus Time-of-Flight Kinect. Comput. Vis. Image Underst..

[19] Ameen W., Al-Ahmari A.M., Hammad Mian S. (2018). Evaluation of Handheld Scanners for Automotive Applications. Appl. Sci..

[20] Kleiner B., Munkelt C., Thorhallsson T., Notni G., Kühmstedt P., Schneider U. (2014). Handheld 3-D Scanning with Automatic Multi-View Registration Based on Visual-Inertial Navigation. Int. J. Optomechatron..

[21] Byczkowski T., Lang J. A Stereo-Based System with Inertial Navigation for Outdoor 3D Scanning. Proceedings of the 2009 Canadian Conference on Computer and Robot Vision.

[22] Ayaz S.M., Danish K., Bang J.-Y., Park S.-I., Roh Y., Kim M.Y. (2015). A multi-view stereo based 3D hand-held scanning system using visual-inertial navigation and structured light. MATEC Web Conf..

[23] Wang X., Xie Z., Wang K., Zhou L. (2018). Research on a Handheld 3D Laser Scanning System for Measuring Large-Sized Objects. Sensors.

[24] Peng T., Zhang Z., Song Y., Chen F., Zeng D. (2019). Portable System for Box Volume Measurement Based on Line-Structured Light Vision and Deep Learning. Sensors.

[25] Deniz C., Cakir M. (2018). A solution to the hand-eye calibration in the manner of the absolute orientation problem. Ind. Robot. Int. J. Robot. Res. Appl..

[26] Park F., Martin B. (1994). Robot sensor calibration: Solving AX = XB on the Euclidean group. IEEE Trans. Robot. Autom..

[27] Fu Z., Pan J., Spyrakos-Papastavridis E., Chen X., Li M. (2020). A Dual Quaternion-Based Approach for Coordinate Calibration of Dual Robots in Collaborative Motion. IEEE Robot. Autom. Lett..

[28] Wang G., Li W.-L., Jiang C., Zhu D.-H., Xie H., Liu X.-J., Ding H. (2021). Simultaneous Calibration of Multicoordinates for a Dual-Robot System by Solving the AXB = YCZ Problem. IEEE Trans. Robot..

[29] Li A.G., Wang L., Wu D.F. (2010). Simultaneous robot-world and hand-eye calibration using dual-quaternions and Kronecker product. Int. J. Phys. Sci..

[30] Cao C.-T., Do V.-P., Lee B.-R. (2019). A Novel Indirect Calibration Approach for Robot Positioning Error Compensation Based on Neural Network and Hand-Eye Vision. Appl. Sci..

[31] Guo Y., Song B., Tang X., Zhou X., Jiang Z. (2020). A measurement method for calibrating kinematic parameters of industrial robots with point constraint by a laser displacement sensor. Meas. Sci. Technol..

[32] Pachtrachai K., Vasconcelos F., Chadebecq F., Allan M., Hailes S., Pawar V., Stoyanov D. (2018). Adjoint Transformation Algorithm for Hand–Eye Calibration with Applications in Robotic Assisted Surgery. Ann. Biomed. Eng..

[33] Zhang Z. (2000). A flexible new technique for camera calibration. IEEE Trans. Pattern Anal. Mach. Intell..

[34] Professional and Industrial PorTable 3D Scanners. https://www.creaform3d.com.cn/zh/bian-xi-shi-3d-sao-miao-yi.

